# P and N type copper phthalocyanines as effective semiconductors in organic thin-film transistor based DNA biosensors at elevated temperatures†

**DOI:** 10.1039/c8ra08829b

**Published:** 2019-01-15

**Authors:** Nicholas T. Boileau, Owen A. Melville, Brendan Mirka, Rosemary Cranston, Benoît H. Lessard

**Affiliations:** University of Ottawa, Department of Chemical and Biological Engineering 161 Louis Pasteur Ottawa Ontario K1N 6N5 Canada benoit.lessard@uottawa.ca

## Abstract

Many health-related diagnostics are expensive, time consuming and invasive. Organic thin film transistor (OTFT) based devices show promise to enable rapid, low cost diagnostics that are an important aspect to enabling increased access and availability to healthcare. Here, we describe OTFTs based upon two structurally similar P (copper phthalocyanine – CuPc) and N (hexdecafluoro copper phthalocyanine – F_16_-CuPc) type semiconductor materials, and demonstrate their potential for use as both temperature and DNA sensors. Bottom gate bottom contact (BGBC) OTFTs with either CuPc or F_16_-CuPc semiconducting layers were characterized within a temperature range of 25 °C to 90 °C in both air and under vacuum. CuPc devices showed small positive shifts in threshold voltage (*V*_T_) in air and significant linear increases in mobility with increasing temperature. F_16_-CuPc devices showed large negative shifts in *V*_T_ in air and linear increases in mobility under the same conditions. Similar OTFTs were exposed to DNA in different hybridization states and both series of devices showed positive *V*_T_ increases upon DNA exposure, with a larger response to single stranded DNA. The N-type F_16_-CuPc devices showed a much greater sensing response than the P-type CuPc. These findings illustrate the use of these materials, especially the N-type semiconductor, as both temperature and DNA sensors and further elucidate the mechanism of DNA sensing in OTFTs.

## Abstract 2

This study illustrates the use of an N-type semiconductor, in both temperature and DNA sensors and further elucidates the mechanism of DNA sensing in OTFTs.

## Graphical abstract

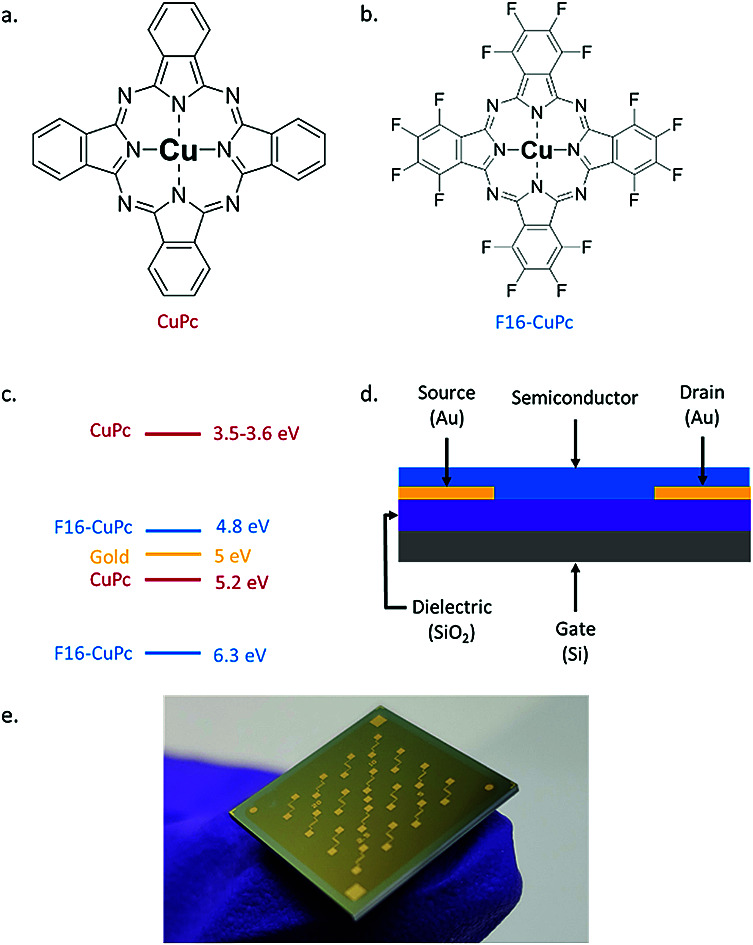

## Introduction

Organic thin films transistors (OTFTs) have shown promise as sensors for detecting various biological analytes such as glucose,^1^ DNA,^2^ thrombin,^3^ bovine serum albumin^4^ and brain injury markers.^5^ OTFTs and their functionally related cousins organic electrochemical transistors (OECTs) are well suited as biological sensors as they can be low-cost, disposable, and mechanically robust.^6–8^ While many OTFT biosensors are not able to match state of the art bioanalytical methods detection limits and sample complexities, they are well positioned to soon serve as rapid and low-cost point of care diagnostics.

DNA sequencing and sensing has rapidly developed since the first human genome was mapped.^9^ While many new technologies have been developed that have increased read times, decreased equipment footprint and lowered cost,^10,11^ there is still a wide margin for improvement. In particular, there are situations in which low cost, high throughput, point of care sensors that do not rely upon amplification would be useful, such as in infectious disease detection.^12,13^ OTFT based DNA detection technology is well suited for these applications due to its potential for high sensitivity^14,15^ and low cost manufacturing.

Currently, various groups have investigated the use of OTFTs as DNA sensors. These devices detect target DNA strands by capturing double stranded DNA (dsDNA) onto the active layer of the OTFT. Physical adsorption of DNA,^16^ electro-immobilization,^17^ and chemical immobilization^18^ have all been investigated for fixing dsDNA, or single stranded DNA (ssDNA) probes, to the surface of an electrode or the semiconductor material itself. ssDNA has a linear structure comprised of four different bases which will bind with a complementary ssDNA strand to form a double helix (dsDNA) that orders the π orbitals of the bases. Upon applying a bias, charge hopping will occur across these bases and thus through the dsDNA.^19,20^ Additionally, each strand has a negatively charged phosphate backbone. When the probe and target ssDNA are captured at the semiconductor surface of an OTFT, there is an increase in negative charge at that surface, due to these phosphates, that can interact with the charge carriers present in the film. This results in a change in the electrical environment of the semiconducting active layer and therefore a measurable change in the OTFT's threshold voltage (*V*_T_), field-effect mobility (*μ*) and/or on/off ratio (*I*_on/off_). For example, Zhang and Subramanian found that upon exposure of a pentacene based OTFT to dsDNA, a positive shift in *V*_T_ of 19.6 V is observed.^2^ Similarly Gui and Wang used pentacene OTFTs with an additional thin layer of CuPc as an interface for DNA to adsorb onto – they observed a positive shift in *V*_T_ of 8 V.^16^ Liu *et al.* exploited the net negative charge of ssDNA to improve sensitivity through an increase in immobilization efficiency by applying a positive bias during the immobilization period on a pentacene OTFT.^17^ In all these reports and others in the literature, the researchers used exclusively P-type semiconductors, such as pentacene, in a variety of OTFT DNA sensor device architectures.^18,21–24^

An aspect of sensor design for most OTFT-based DNA sensors that has been overlooked is the required elevated temperature for DNA binding. To ensure specific binding of DNA, either at the point of detection or before, the strands must be in solution at a specific setpoint below the melting temperature (*T*_M_) of the particular DNA sequences being investigated.^25–27^ Therefore it is imperative that the OTFT devices be operated at elevated temperature (typically optimal binding occurs between 40 °C to 70 °C depending on the particular DNA sequence) to ensure specific DNA binding and to reduce non-specific binding. Typically, a temperature of *T*_M_ – 5 °C is considered optimal for specific binding of complementary DNA strands. Currently only Gui *et al.* have investigated elevated temperature operation of OTFT DNA sensors.^28^ They investigated the effect of hybridization times at *T* = 20 °C, 45 °C and 60 °C on sensor sensitivity. For their particular DNA sequence, they found that optimal sensor response occurred at 45 °C (13 °C below their sequence *T*_M_). To the best of our knowledge there are no reports of OTFT-based DNA sensors which utilize N-type semiconductors or that operate just below the *T*_M_ value of the DNA analytes they are using.

Studies on the effect of operating temperature on the performance of organic semiconductors are uncommon,^29–34^ and the majority of the studies that have been conducted are utilizing P-type semiconductors such as pentacene. An improved and varied understanding of temperature effects on different charge carriers in OTFTs could lead to them being used in a myriad of important applications. For example, many medical therapies require precise temperature control at a patient surface, such as in hypothermia therapy,^35,36^ laser therapy,^37^ and cryosurgery.^38^ Often, IR thermography is used to measure patient surface temperature, but unfortunately this can be inaccurate, especially when various topical substances are used during treatment such as ultrasound gel.^39^ Temperature sensing capabilities would also be useful in such applications as electronic skin, and smart fabrics. Most studies on varied OTFT operating temperature can be divided between low temperature (below room temperature), and high temperature (above room temperature) investigations. Many studies focusing on low temperature operation are doing so to investigate charge conduction models and factors affecting OTFT response to variable temperature operation. For example, Lin & Hung found that in pentacene OTFTs *V*_T_ increases positively with increasing temperature between *T* = −150 °C and *T* = 25 °C, a result they hypothesize is due to deep hole trapping.^29^ Similarly, Sun *et al.* found that in the temperature range *T* = −213 °C to *T* = 17 °C pentacene OTFTs threshold voltage increases linearly with increasing temperature and that humidity has significant effects on the number of deep hole traps.^30^ Only a single study by Chesterfield R. J. *et al.* has investigated the performance of an N-type semiconductor (PTCDI-C_5_) in OTFTs at variable low temperature operation (*T* = −173 °C to *T* = 25 °C) and it was found to have a thermally activated mobility.^32^ Finally, some groups have investigated elevated temperature operation of OTFTs and the use of OTFTs as temperature sensors. Jung *et al.*, among others, have investigated the use of pentacene based OTFTs at temperatures ranging from 0 °C to 90 °C and have observed measurable changes in the source/drain current (*I*_DS_), *μ*, and *V*_T_.^34,40,41^ Increasing temperature above room temperature in air led to decreased mobility, a more positive *V*_T_, and increased off current in two P-type semiconducting polymers. This was hypothesized to occur due to polymer oxidation and gas adsorption.^31^ Most successfully, Ren *et al.* constructed a pentacene based flexible temperature sensor, measuring changes in *I*_DS_, and enhancing sensitivity by incorporating a silver nanoparticle layer.^33^ To the best of our knowledge, no reports have explored the investigation of N-type materials in OTFTs above 25 °C.

**Fig. 1:**
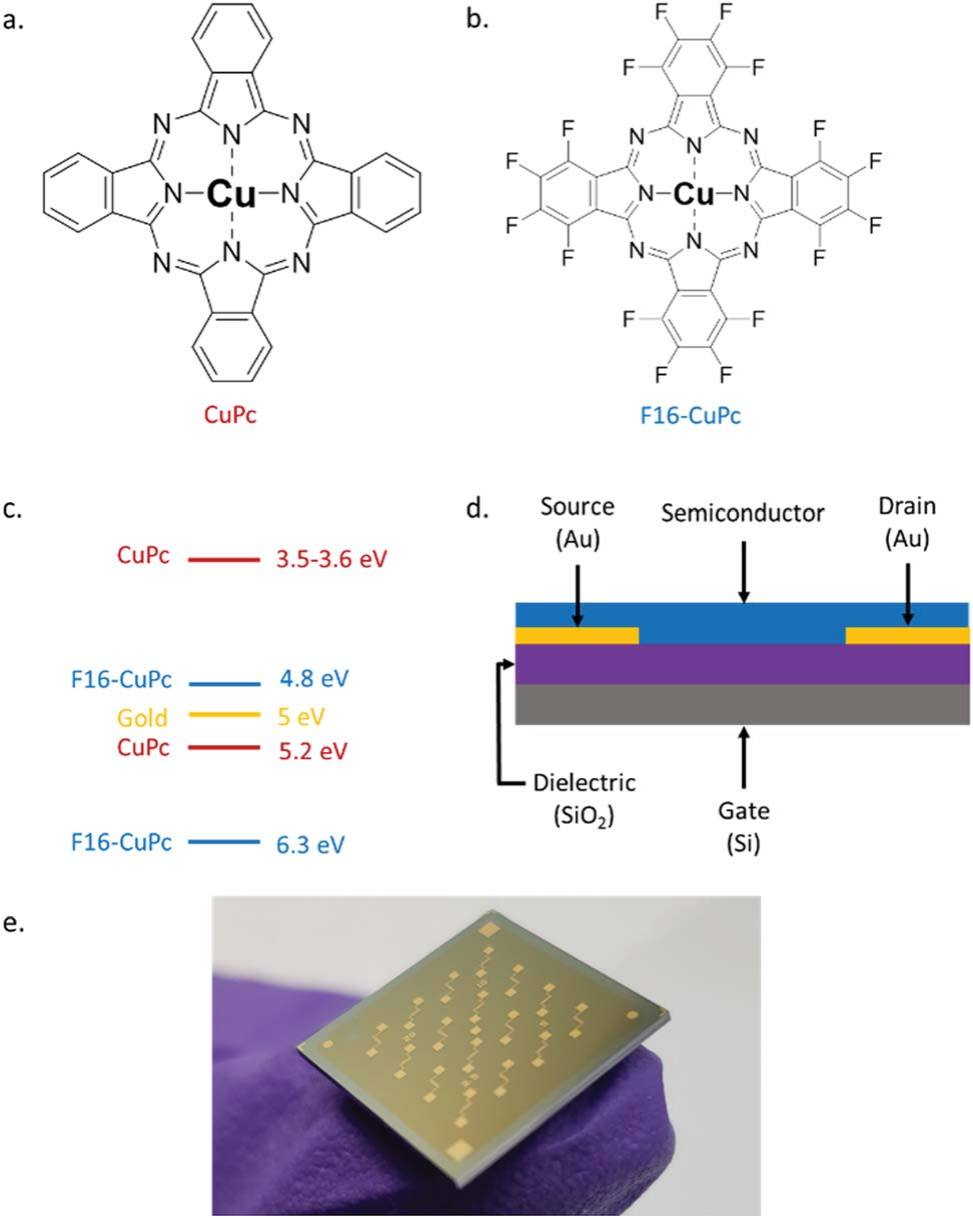
(a) Structure of copper phthalocyanine (CuPc) (b) structure of copper hexadecafluorophthalocyanine (F_16_-CuPc). (c) HOMO/LUMO levels of CuPc^43,44^ and F_16_-CuPc^45^ relative to the work function of gold (yellow). (d) Bottom gate bottom contact organic thin transistor (OTFT) structure. (e) Picture of one actual Fraunhofer device.

In this study, we report the first OTFT DNA sensors using an N-type semiconductor, copper hexadecafluorophthalocyanine (F_16_-CuPc) (Fig. 1b), and compare it to a structurally similar P-type material: copper phthalocyanine (CuPc) (Fig. 1a). We also investigate the effects of ambient temperature (between 25 °C to 90 °C) operation of OTFTs constructed with each of the semiconducting materials in both air and vacuum (*P* < 0.1 Pa). Both CuPc and F_16_-CuPc are robust materials that make air-stable OTFTs that have been extensively researched and characterized,^42^ thus making good material candidates for investigation. As the negatively charged DNA backbone can affect the positive and negatively charged carriers differently, resulting devices are expected to have distinct operational responses which could then be detected separately or utilized together to enhance sensitivity/selectivity in a DNA sensor.

## Results and discussion

### Effect of OTFT operation temperature

**Fig. 2:**
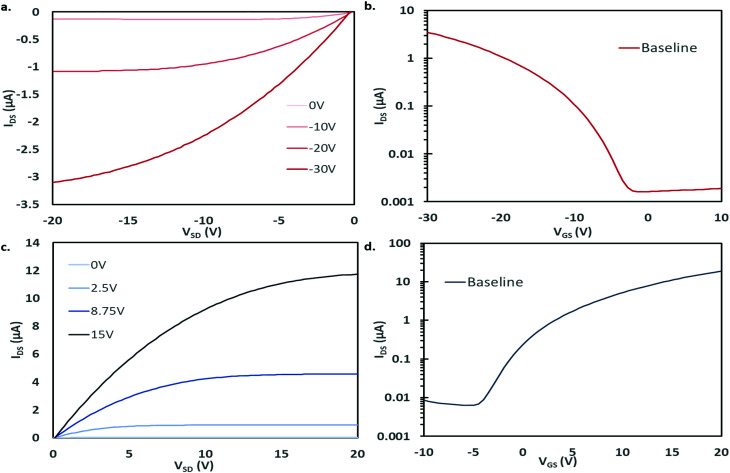
Output and transfer curves for baseline BGBC OTFTs tested at 25 °C. (a) Output curve and (b) transfer curve (*V*_DS_ = −20 V) for CuPc devices. (c) Output curve and (d) transfer curve (*V*_DS_ = 20 V) for F_16_-CuPc devices.

Bottom gate, bottom contact (BGBC) OTFTs with an interfacial trichloro(octyl)silane (OTS) layer were fabricated by thermal vacuum deposition with either CuPc or F_16_-CuPc as the semiconducting layer on heavily doped silicon substrates with a thermally grown silicon dioxide dielectric. The typical output and transfer curves for baseline devices characterized at 25 °C in air are shown in Fig. 2a–d for both CuPc and F_16_-CuPc.

**Fig. 3:**
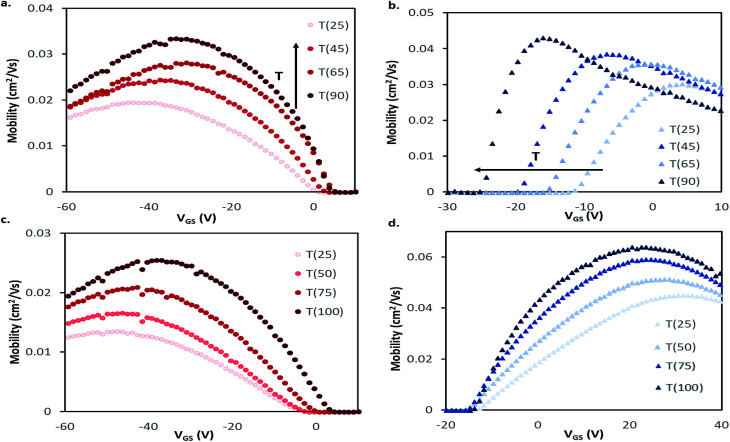
Field-effect mobility for (a and c) CuPc and (b and d) F_16_-CuPc BGBC devices deposited at *T* = 140 °C with respect to applied gate-source voltage (*V*_GS_) for characteristic devices at varied temperatures in air (a and b) and vacuum (c and d). This mobility was calculated between adjacent points in the transfer data using eqn (2). Devices were tested in the range of *T* = 25 °C to *T* = 90 °C in air, and *T* = 25 °C to *T* = 100 °C in vacuum.

Our research group has recently found significant changes in P-type small molecules when tested in air compared to vacuum (*P* < 0.1 Pa)^46^ and demonstrated differences in temperature response under these conditions for two P-type semiconducting polymers.^31^ Therefore, we characterized these CuPc and F_16_-CuPc devices at discrete temperatures ranging from *T* = 25 °C to *T* = 90 °C in air and from 25 °C to *T* = 100 °C in vacuum. Due to equipment heating limitations, we were unfortunately not able to test at temperatures greater than 90 °C in air. As temperature increased in air a noticeable increase in hole mobility (*μ*_H_) and a slight positive shift in *V*_T_ was observed for CuPc. This change is well illustrated in Fig. 3a as a shift in the positive (+) direction and up in the *μ*_H_ as a function of gate voltage (*V*_GS_) graph. For F_16_-CuPc devices, a large negative shift in *V*_T_ and a small increase in electron mobility (*μ*_E_) were observed (Fig. 3b). To determine the impact of environment, identical devices that had never been exposed to air were characterized under vacuum. The resulting plots can be found in Fig. 3c and d for CuPc and F_16_-CuPc, respectively. The low pressure environment slightly improved the F_16_-CuPc devices, increasing *μ*_E_, while the opposite was true for CuPc devices. From this baseline, similar changes in *V*_T_ and *μ*_H_ were observed with increasing temperature for CuPc as in air. F_16_-CuPc, on the other hand, lacked the large negative *V*_T_ shift observed in air with increasing temperature, demonstrating primarily a small increase in *μ*_E_ as with CuPc under both conditions.

**Fig. 4:**
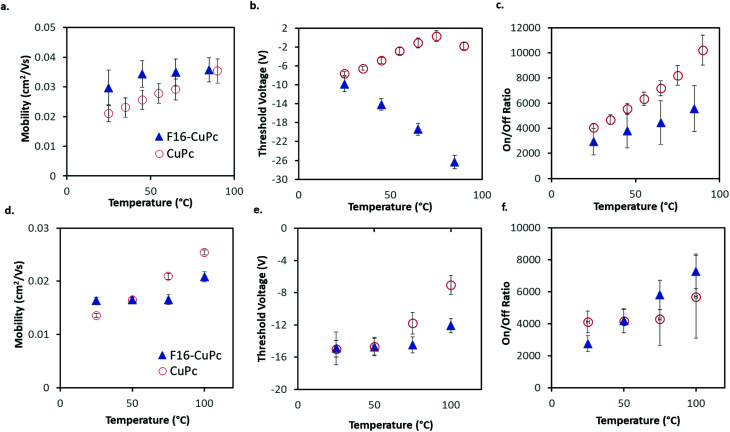
Performance of BGBC CuPc and F_16_-CuPc devices (deposited at *T* = 140 °C) in air (a, b and c) or vacuum (d, e and f) at various temperatures. (a and d) Field-effect mobility. (b and e) Threshold voltage. (c and f) On/off ratio. Presented are the averages for four devices with error bars representing the standard deviation. The legend in (a and d) is the same as in (b and e) and (c and f). Devices were tested in the range of *T* = 25 °C to *T* = 90 °C in air, and *T* = 25 °C to *T* = 100 °C in vacuum.

The average *μ*_H_ in CuPc devices in air increased by about 1% °C^−1^ increase in temperature from 25 °C to 90 °C with a coefficient of determination of *R*^2^ = 0.995 (Fig. 4a). In the same temperature range, *V*_T_ shifted from about −7.6 V to −1.8 V, which correlates to a change of about 0.11 V °C^−1^. This value in the units of V °C^−1^ can be defined as the sensitivity of the device as it shows how much the output value (V) changes with changing input that is being measured (°C), this also holds for the mobility % change per °C values. The change in μ_e_ of F_16_-CuPc based OTFTs was smaller, about 0.1% °C^−1^ with *R*^2^ = 0.785. However, F_16_-CuPc based OTFTs experienced a significant change in *V*_T_ between *T* = 25 °C and 90 °C as seen in Fig. 4b. A *V*_T_ shift was measured from −9.9 V to −26.4 V, correlating to a shift of −0.25 V °C^−1^. As expected, the *I*_on/off_ ratios for both materials changed similarly to the changes in mobility. Similar effects were observed under vacuum (Fig. 4d–f) except F_16_-CuPc devices appeared to have little or no significant change in *V*_T_.

Identical experiments were performed on devices that were fabricated through thermal evaporation on heated substrates (*T* = 25 °C), which is significantly cooler then those described above which were fabricated with substrates heated to 140 °C during deposition. The results obtained from these devices can be seen in Fig. S1 and S2.† These devices displayed similar trends except for a shift in *V*_T_ for the F_16_-CuPc devices in air that was not as large, Δ*V*_T_ = 12.1 V (it shifted with a rate of 0.19 V °C^−1^).

For the P-type CuPc, the small positive shift in *V*_T_ is similar to what has been reported for pentacene.^34^ This shift in *V*_T_ could be explained by a decreased number of positive carriers being confined to a trap state at higher temperature, as charge carriers have been shown to be affected by thermal activation.^41^ The increase in mobility with temperature is known to occur as the generally accepted charge conduction mechanisms of OTFTs are temperature depedent.^32,47^ The same mechanism might explain the increase in electron mobility with rising temperature observed for F_16_-CuPc in both air and vacuum. However, a large negative change in *V*_T_ with increasing temperature is observed in air, shifting the devices to an “on” state without a gate bias. Since the shift is not observed in vacuum, it is likely caused by some component of air such as oxygen or water. Changes in *V*_T_ can be attributed to energetically deep traps, or gate bias stress effects,^32^ with negative changes associated with hole traps or accumulation of positive charge. Typically, oxygen suppresses electron transport and can act as a hole dopant in some materials, so it is difficult to explain how it might shift the operating bias negatively. It has been reported that devices with SiO_2_ dielectrics have positive threshold voltage shifts upon exposure to ambient air due to water interacting with dangling –OH groups at the insulator surface.^48^ Thus, it's possible that upon increasing the temperature of the devices, water desorption is shifting the threshold voltage of the devices negatively. Although this phenomena would affect both CuPc and F_16_-CuPc devices, the differing chemical structure or morphology of the semiconductors may explain the differing response however further investigation is warranted.

**Fig. 5:**
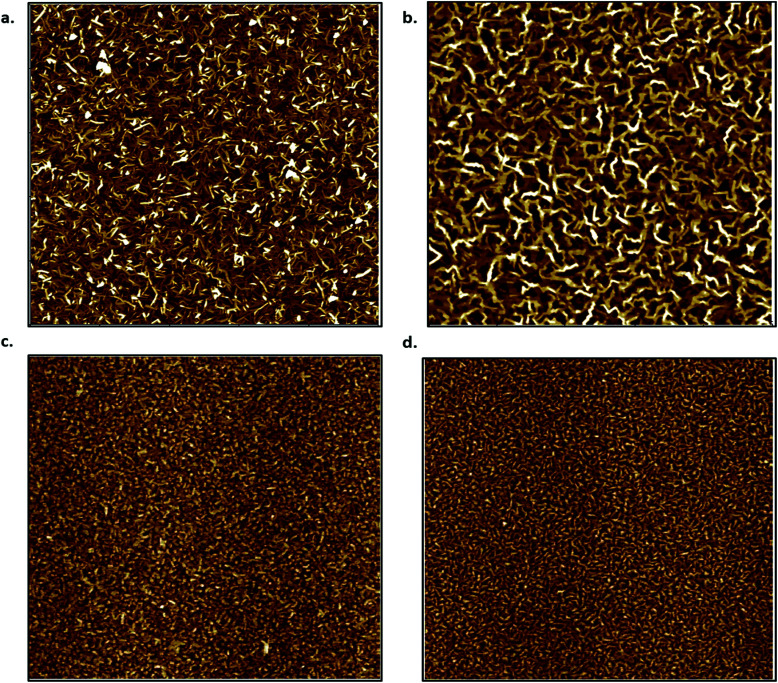
AFM images of CuPc (a and c) and F_16_-CuPc (b and d) thin films deposited on SiO_2_ substrates at 140 °C (a and b) and 20 °C (c and d). Images (a and b) are 5 μm × 5 μm while (c and d) are 2.5 μm × 2.5 μm.

To investigate film morphology further, AFM images were taken of 15 nm films of both CuPc and F_16_-CuPc deposited at both *T* = 140 °C and *T* = 25 °C. These images can be seen in Fig. 5. The above films were found to have root mean square (*R*_q_) values of 1.84 nm, and 1.21 nm for high temperature (140 °C) deposited CuPc and F_16_-CuPc films respectively. The low temperature (25 °C) films had *R*_q_ values of 1.93 nm (CuPc) and 1.53 nm (F_16_-CuPc). Thus, the F_16_-CuPc are smoother in both cases, while the smoothest films were obtained at the higher deposition temperatures. It is also apparent that high temperature deposition of F_16_-CuPc (Fig. 5b) films have larger grains than the CuPc films (Fig. 5a). Grain sizes between the low temperature films are difficult to distinguish by visual inspection (Fig. 5c and d). Between the two F_16_-CuPc samples, the smoother sample and the sample with the larger grain size (Fig. 5b) experience a larger Δ*V*_T_ over the temperature range. This is also true for the CuPc samples, although the Δ*V*_T_ is in the positive direction. These changes in surface area for gas adsorption appear to reflect a greater electronic sensitivity towards temperature in air.

These results discussed illustrate that both CuPc and F_16_-CuPc based OTFTs can operate between *T* = 25 °C and 90 °C with controlled and predictable changes in performance as a function of operating temperature. This indicates that both molecules could function as components of OTFT-based temperature sensors.

### DNA hybridization sensing

**Fig. 6:**
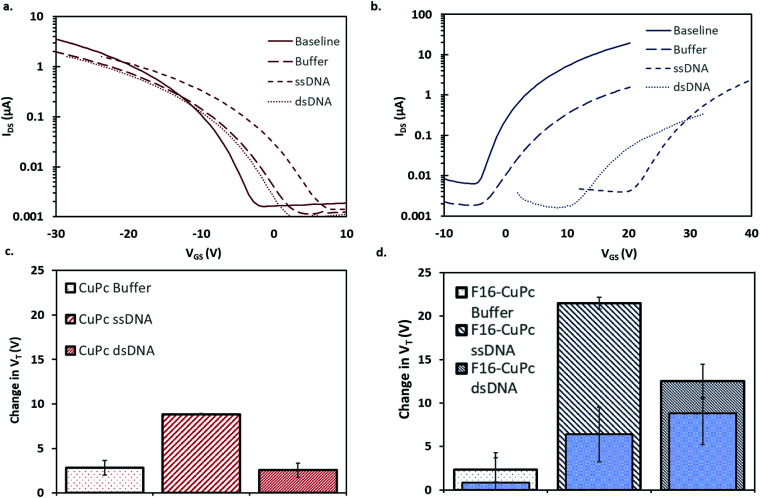
Transfer curves (a and b) and absolute change in threshold voltage (c and d) of 1 μL of 1 μM ssDNA, 1 μM dsDNA or buffer were added to baseline of CuPc OTFT (a and c) and F_16_-CuPc (b and d). All characterization was performed at 51.1 °C in air except for the superimposed inner bars in (d) (filled blue rectangle) which were characterized at 25.0 °C.

To investigate DNA hybridization sensing with OTFTs, a series of analytes were pipetted onto either CuPc or F_16_-CuPc based devices. For each of these analytes, the base device was first characterized, followed by the analyte deposition, rinsing with deionized water, drying under vacuum for 3 minutes, and then re-characterized. During this series of experiments, the OTFTs were operated at 51.1 °C or 25.0 °C. The elevated temperature is equal to *T*_M_ – 5 °C for the specific complementary probe and target DNA sequences used. As outlined, this is important to ensure specific and efficient hybridization of the complementary strands. If the temperature at which binding occurs is too high, then binding will not occur due to it being thermodynamically unfavourable. If the temperature is too low, then unspecific binding can occur. This procedure facilitated the comparison of affects across different analytes on the devices. The charge mobilities for both CuPc and F_16_-CuPc decreased between 60–70% from their initial values no matter the analyte added. There was no statistically significant difference between buffer, ssDNA, and dsDNA; suggesting the buffer and the procedure itself impacts or degrades the semiconducting material/structure and function. As such, the on/off ratios for each analyte also decreased but with no significant differences between these analytes. However, the change in *V*_T_ values did vary significantly between different analytes. Fig. 6a and b show the changes in the transfer curve for CuPc and F_16_-CuPc respectively. These curves illustrate clear shifts in *V*_T_ as well as changes in *μ* and *I*_DS_ with exposure to different DNA analytes but the difference between ssDNA and dsDNA were determined as statistically insignificant. At 51.1 °C, an average positive shift of *V*_T_ = +2.8 V (from baseline) was observed for CuPc based OTFT after buffer addition and an average positive shift of *V*_T_ = +8.8 V (from baseline) after ssDNA addition and *V*_T_ = +2.6 V after dsDNA addition (from baseline) (Fig. 6c). Similarly to CuPc, F_16_-CuPc based OTFTs also experienced a positive shift compared to baseline Δ*V*_T_ = +2.3 V, +21.5 V and +12.5 V with the addition of buffer, ssDNA and dsDNA, respectively. Additionally, the exact same experiments were performed on F_16_-CuPc devices but operated at 25.0 °C. This is seen in the inset bars of Fig. 6d. The threshold voltage shifts of ssDNA and dsDNA were *V*_T_ = +6.4 V, and +8.8 V, respectively, but were determined as statistically insignificant.

Similarly to what is observed for CuPc devices, reports employing pentacene as a semiconductor also found small positive *V*_T_ shifts and decreases in *I*_DS_ with the addition of ssDNA.^49,50^ Gui *et al.* reported larger *V*_T_ shifts around 8 V for a pentacene device that utilized a thin CuPc layer that acted as an adsorption site for DNA as well as environmental protection for the pentacene semiconducting layer.^16^ A positive shift in *V*_T_ is often associated with electron trapping, but more directly it is associated with the accumulation of negative charges which would electrostatically facilitate the injection of holes and oppose that of electrons.^51^ Thus, positive changes in *V*_T_ upon addition of DNA could be explained by the negative charge associated with the phosphate groups in the DNA backbone. The addition of dsDNA to CuPc OTFTs resulted in a positive shift of *V*_T_ = +2.6 V (from baseline) which is much less than the increase seen with ssDNA, *V*_T_ = +8.8 V (Fig. 6c). At surfaces, ssDNA has a much higher effective density than dsDNA due to its more flexible structure.^52^ This difference is due to the relative chain rigidity and intermolecular coulombic repulsions of dsDNA.^53^ The higher chain density for ssDNA might lead to a higher charge density at the surface of the semiconducting material compared to dsDNA, regardless of dsDNA having double the negative charge per molecule. This might explain the larger threshold voltage shift observed for ssDNA compared to dsDNA.

F_16_-CuPc based OTFTs also experienced a positive shift compared to baseline. These results, shown in Fig. 6b and d, again show a significant difference in Δ*V*_T_ between ssDNA and dsDNA, suggesting reduced charge density at the semiconductor surface for dsDNA analytes. A larger Δ*V*_T_ is seen in F_16_-CuPc than for CuPc. This mirrors the larger response to temperature seen in F_16_-CuPc in air, although the threshold voltage shift is in the opposite direction. Perhaps the film morphology or energetics of the N-type F_16_-CuPc make it more susceptible to electron trapping upon addition of various aqueous analytes. The larger grains, and seemingly less dense film, may be more permeable to strands of DNA, increasing the relative changes observed.

Finally, as seen in the superimposed inner bars in Fig. 6d, the sensing response between ssDNA and dsDNA when sample injection and device operation is at *T* = 25 °C, is not significantly different. This is expected as DNA requires an elevated temperature approaching its *T*_M_ to bind specifically. Thus it's expected that the two samples are in similar binding states even though one sample contains complementary DNA (dsDNA label) while the other does not (ssDNA label). Of note, is that the ssDNA response at lower temperature is much lower than the ssDNA response at higher temperature. Thus it appears that higher temperature device operation alone could increase sensing response for DNA. This could be due to differing analyte interactions at the surface of the film at different operation temperatures.

Lastly, we investigated the effect of dsDNA concentration on the change in *V*_T_ for F_16_-CuPc based OTFTs. It was found that the effective concentration range of the F_16_-CuPc sensor is at least between 0.01 μM and 0.1 μM, and as the concentration of DNA increases, so too does the magnitude of the change in *V*_T_ within the specified range. Further experiments at various concentrations is required to establish the full operating window, including the limit of detection. Further steps could be necessary to maximize this operating window, such as modifying the electrode design, channel geometry or materials. Regardless, these results illustrate that N-type semiconductors such as F_16_-CuPc can be utilized as the sensor element for dsDNA through simple physical adsorption.

## Experimental

### Materials

Copper phthalocyanine (CuPc, 90%), and copper(ii) 1,2,3,4,8,9,10,11,15,16,17,18,22,23,24,25-hexadecafluoro-29*H*,31*H*-phthalocyanine (F_16_-CuPc, >99.9%) were obtained from TCI Chemicals. CuPc was purified twice by train sublimation before use. All chemicals were used as received unless otherwise specified. The following single stranded deoxyribonucleic acid (DNA) oligonucleotides were purchased from Integrated DNA Technologies.

**Table:** 

DNA	Sequence (5′– 3′, 20bp)	*T*_M_ °C^a^
Probe	CAC ACG GAA CTG AAC AAG GTC	56.1
Target (complementary to probe)	GAC CTT GTT CAG TTC CGT GTG	56.1
Random control	GAG TCT TAA TAA GAA TGC ATC	46.3
^a^ *T*_M_ = melting temperature of DNA strand.		

The DNA oligonucleotides were resuspended in water to a concentration of 100 μM. The DNA was aliquoted and frozen until use. DNA solutions were made to the desired concentration for each experiment with 5× saline-sodium citrate (SCC) buffer/0.1% Tween-20. A stock 20× SCC solution consisting of 3 M NaCl, 300 mM trisodium citrate, with pH adjusted to 7 with HCl was used.

### Preparation of devices

Heavily n-doped silicon substrates with a 230 nm SiO_2_ dielectric and prefabricated gold source–drain electrodes from Fraunhofer IPMS (*W* = 2000 μm, *L* = 20 μm) were washed with acetone and dried with nitrogen. They were then treated with oxygen plasma for 15 minutes to clean and hydrolyze the surface. Substrates were then rinsed with water and isopropanol, before a 1 hour surface treatment in 1% v/v octyltrichlorosilane (OTS) in toluene at 70 °C. Silane-treated substrates were washed with toluene and isopropanol and dried for 1 hour at 70 °C under vacuum. CuPc and F_16_-CuPc were deposited using physical vapor deposition in an Angstrom EvoVac thermal evaporator with a target thickness of 150 Å and a rate of 0.3 Å s^−1^ at 140 °C. Heated substrates were allowed to cool to room temperature before being removed from the vacuum chamber, usually overnight.

### OTFT testing & electrical characterization

Contact with the source-drain electrodes was made with BeCu alloy probe tips. Output curves were obtained by fixing the gate voltage (*V*_GS_) at discrete values and sweeping the source–drain voltage (*V*_SD_). Electrical measurements were performed using a custom electrical probe station with a chamber allowing for controlled atmosphere, oesProbe A10000-P290 (Element Instrumentation Inc. & Kreus Design Inc.) with a Keithley 2614B to control source–drain voltage (*V*_DS_), gate voltage (*V*_GS_), and measure source–drain current (*I*_DS_). *V*_DS_ was held constant while *V*_GS_ was varied to obtain measurements of *I*_DS_. From these measurements, saturation region field-effect mobility, on/off current ratio, and threshold voltage were determined. The general expression relating current to field-effect mobility and gate voltage in the saturation mode is given in eqn (1):1
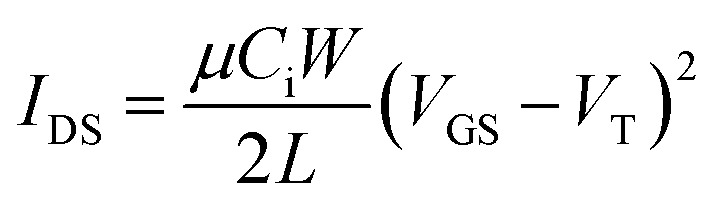
where *I*_DS_ is the source–drain voltage, *μ* is the field-effect mobility of the particular material, *C*_i_ is the capacitance density, *W* is the width of the channel, *L* is the length of the channel, *V*_GS_ is the gate–source voltage, and *V*_T_ is the threshold voltage. To obtain a linear relation, the square root of eqn (1) is taken, giving eqn (2), so that the mobility and threshold voltage can be calculated directly from the slope and *x*-intercept of an 
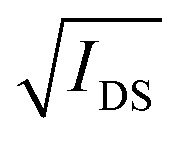
*vs. V*_GS_ curve.2
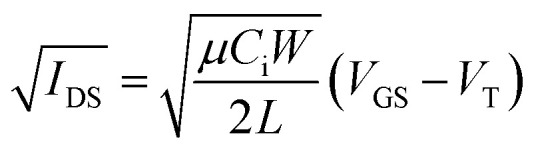


Finally, the on/off ratio is determined by eqn (3):3
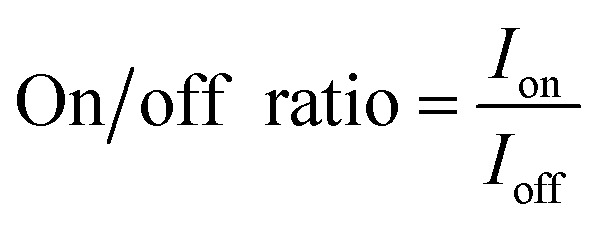
where *I*_on_ and *I*_off_ are the highest and lowest currents, respectively, measured in the characterized gate voltage range.

### DNA experiments

During testing of the devices used in DNA sensing experiments, the devices were operated at 51.1 °C (as this is the melting temperature (*T*_M_ – 5 °C) or 25 °C. The devices were tested with no analytes to establish a baseline at 51.1 °C, then either 2 μL of buffer, or one of the DNA solutions were pipetted directly onto the source/drain channel. Then, the droplets were left to evaporate (2 minutes), then the devices were rinsed with deionized water. They were then dried with nitrogen and placed under vacuum for 3 minutes. The device was then retested. DNA solutions were made by first heating the individual ssDNA to 95 °C for 15 seconds, then either mixing the probe and target strands (complementary) or the probe and control strands (non-complementary) in the desired concentration, and then pipetting onto the transistor surface.

### AFM

A Park NX10 system was used in non contact mode with a PPP-NCH-20 tip, with a scan rate of 0.7 Hz and image size of 512 × 512 pixels. The images were produced with the XEI software version 1.8.2.

## Conclusions

Bottom gate bottom contact OTFT temperature and DNA sensors were fabricated using both CuPc or F_16_-CuPc as the P or N type semiconductor layer, respectively. Within a temperature range of 25 °C to 90 °C CuPc devices in air showed little change in *V*_T_ but significant and linear increases in mobility. Under the same conditions, F_16_-CuPc showed a linear and significant negative change in *V*_T_, with an increase in mobility as well. Under vacuum, devices with both materials varied similarly with increasing temperature, exhibiting almost no change in *V*_T_ and an increase in mobility. Both CuPc and F_16_-CuPc devices responded differently when treated with ssDNA *versus* dsDNA. The negative charge originating from DNA effects shifted the *V*_T_ in the positive direction for both P and N type materials, with a greater shift observed for ssDNA compared to dsDNA. While similar observations have been reported for P-type semiconductors, detection using an N-type organic semiconductor is unprecedented, more sensitive, and further supports one of the proposed mechanisms for dsDNA detection by OTFTs. Hybrid detection could be possible by examining the changes in *V*_T_ for each material and future efforts will focus on amplification of signal response through device engineering and material selection.

## Conflicts of interest

There are no conflicts to declare.

## Supplementary Material

RA-009-C8RA08829B-s001
