# Supplementary material for: P and N type copper phthalocyanines as effective semiconductors in organic thin-film transistor based DNA biosensors at elevated temperatures
Source: RSC Adv. 2019 Jan 15;9(4):2133–42. doi: 10.1039/c8ra08829b (PMC9059718; doi:10.1039/c8ra08829b)
Supplement: RA-009-C8RA08829B-s001 [file RA-009-C8RA08829B-s001.pdf]

Electronic Supplementary Material (ESI) for RSC Advances.
This journal is © The Royal Society of Chemistry 2019

P and N type copper phthalocyanines as effective semiconductors in organic thin-film transistor based DNA biosensors at elevated temperature

*Nicholas T. Boileau, Owen A. Melville, Brendan Mirka, Rosemary Cranston, Benoit H. Lessard**

University of Ottawa, Department of Chemical and Biological Engineering

161 Louis Pasteur, Ottawa, Ontario, K1N 6N5

*Corresponding Author. E-mail: benoit.lessard@uottawa.ca

Supplementary Information

Figures S1 and S2 display the electronic data for CuPc and F16-CuPc devices deposited at T = 25 °C. These devices were tested identically to those deposited at T = 140 °C.

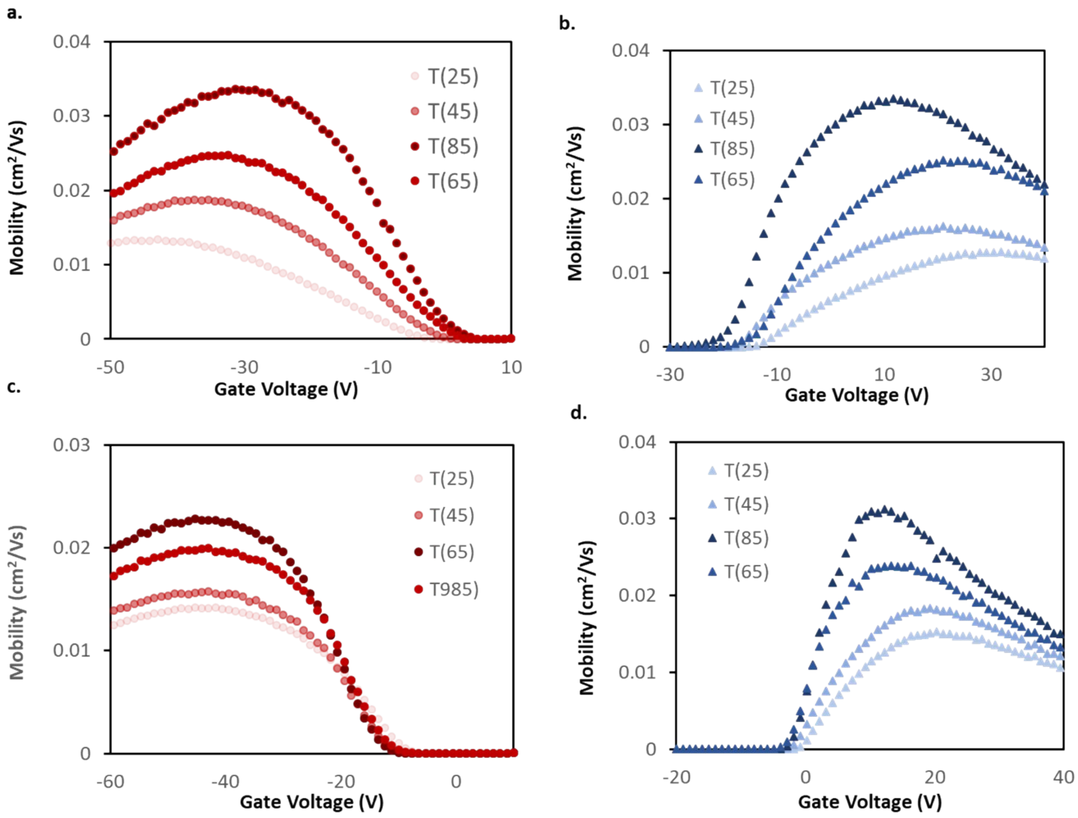

Figure S1. Field-effect mobility for (a,c) CuPc and (b,d) F16-CuPc devices deposited at T = 25 °C with respect to applied gate-source voltage (V_{GS}) for characteristic devices at varied temperatures in air (a, b) and vacuum (c, d). This mobility was calculated between adjacent points in the transfer data using equation 2

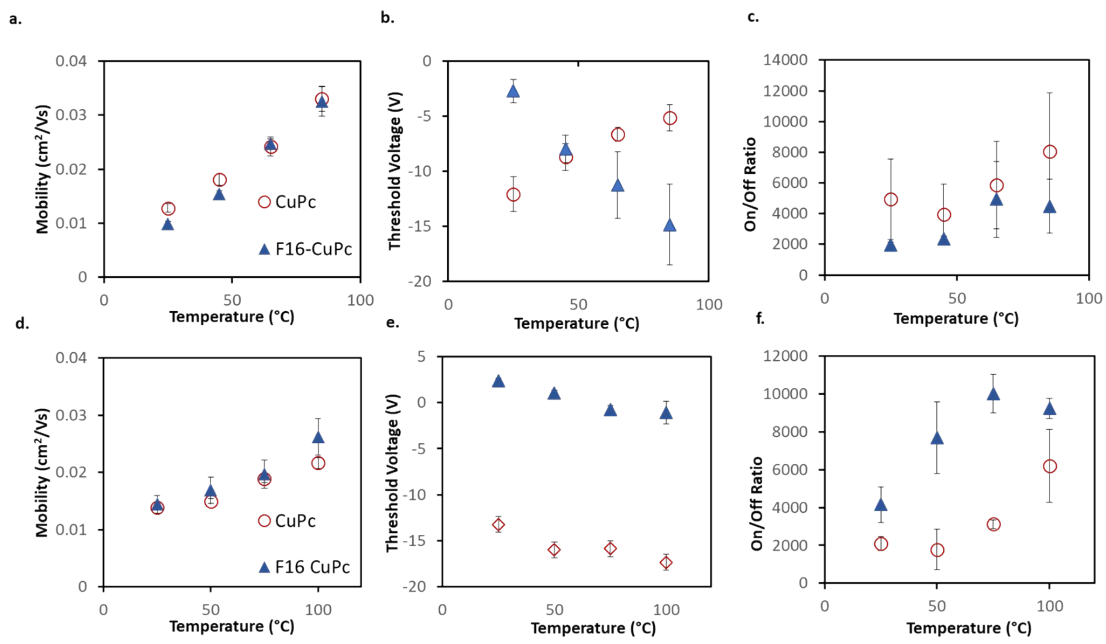

Figure S2. Performance of CuPc and F_{16}-CuPc devices (deposited at T = 140 °C) in air at various temperatures. (a) Field-effect mobility. (b) Threshold voltage (V_T). (c) on/off ratio. Presented are the averages for four devices with error bars representing the standard deviation. The legend in (a) is the same as in (b) and (c).
